# PARP6 inhibition as a strategy to exploit centrosome clustering in cancer cells?

**DOI:** 10.18632/oncotarget.26599

**Published:** 2019-01-22

**Authors:** Zebin Wang, Shaun E. Grosskurth, Huawei Chen

**Affiliations:** Huawei Chen: Bioscience, Oncology, IMED Biotech Unit, AstraZeneca, Boston, USA

**Keywords:** PARP6, multipolar spindle, centrosome clustering, Chk1, AZ0108

Centrosomes function as the main microtubule-organizing centers (MTOCs) and play an essential role during cellular mitosis. As such, centrosome number is tightly controlled, with centrosomes duplicated only once per cell cycle [1]. Unsurprisingly, centrosome abnormality, in particular centrosome amplification, has been frequently observed in human malignancies and is linked with aneuploidy and tumorigenesis. Recently the causative role of centrosome amplification in cancer has been demonstrated in a mouse model [2]. The resulting extra centrosomes from amplification pose a challenge for the proper execution of mitosis since multipolar mitosis is often detrimental to dividing cells and can lead to cell death [3]. Cancer cells, however, have developed tactics to avoid deleterious spindle multipolarity during mitosis through mechanisms such as centrosome clustering, a process that involves assembling multiple centrosomes into pseudo-bipolar spindles and thereby promotes tumor survival [4]. In recent years, a therapeutic strategy to pharmacologically impair centrosome clustering has emerged and demonstrated early promise in the preclinical setting: the goal is to exploit the vulnerability of cancer cells in handling spindle multipolarity by inducing centrosome de-clustering and achieve selective killing of cancer cells while sparing normal tissues.

We recently reported the involvement of mono-ADP-ribosyltransferase PARP6 in maintaining centrosome integrity during mitosis by demonstrating that pharmacological inhibition of PARP6 induced multipolar spindle formation and elicited therapeutic effects in breast cancer [5]. This study was largely enabled by the AstraZeneca collection of PARP inhibitors consisting of mostly phthalazinone and quinazolinone based NAD^+^ mimetic cores that display differential selectivity towards poly- and mono-ADP-ribosyltranferases [6]. Previous siRNA screens implicated PARP activity in centrosome clustering and bipolar spindle formation as a specific mitotic susceptibility in cancer cells [7]. We leveraged this knowledge and conducted parallel biochemical screens against multiple PARP enzymes together with cell-based phenotypic profiling in cancer cells to find that only PARP6 inhibition could drive multipolar spindle formation, with a strong correlation (*R*^2^ = 0.76) observed between PARP6 enzymatic inhibition and multipolar spindle induction potency. We recapitulated the mitotic phenotype with siRNA-based PARP6 knockdown. Together, these results link the suppression of PARP6 enzymatic activity to the induced multipolar spindle centrosome phenotype. We further showed that targeting PARP6 with the optimized small molecule inhibitor AZ0108 effectively induced spindle multipolarity and demonstrated promising anti-tumor activity in various breast cancer models *in vitro* and *in vivo*.

To gain mechanistic understanding of the mitotic phenotype associated with PARP6 inhibition, we sought to identify PARP6 enzyme substrates using a powerful high-content ProtoArray screen consisting of 9,000+ full length human proteins. Centrosome related proteins were enriched as screening hits, including Chk1, which was further confirmed as a specific PARP6 substrate by *in vitro* enzymatic assays and mass spectrometric analysis in cells. Beyond its role in mediating DNA damage responses, Chk1 is also involved in regulating cell-cycle checkpoints such as mitosis. During undisturbed cell cycle, Chk1 was reported to localize to interphase centrosomes and modulate cyclinB-Cdk1 activity, thereby impacting centrosomic mitotic events [8]. Our experiments demonstrated that intracellular ADP-ribosylation of Chk1 accumulated upon mitotic arrest and was abolished by treatment with PARP6 inhibitor AZ0108. Intriguingly, suppressing PARP6 using AZ0108 resulted in marked elevation of Chk1 phosphorylation at S345 and a dose-dependent increase in centrosome-associated Chk1 expression. These events were accompanied by diminished activation of mitotic proteins such as FoxM1, histone H3 and Aurora kinases. Together, these observations support a novel mechanism for PARP6 in regulating Chk1 activation in mitosis *via* ADP-ribosylation and provide a mechanistic basis for multipolar spindle induction through PARP6 inhibition.

**Figure 1:**
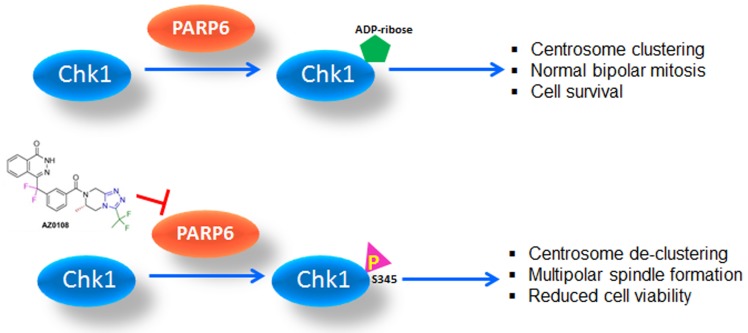
PARP6 as a novel molecular target to exploit cancer cell vulnerability of pseudo-bipolar mitosis for therapeutic benefit

In summary, our study established PARP6 as a novel molecular target for therapeutic intervention in breast cancer and delineated its role in maintaining centrosome integrity *via* modulating Chk1 activity (Figure 1). We also reported the discovery of novel PARP6 substrates and characterized the activity of the first reported PARP6 inhibitor AZ0108 in breast cancer cells. These findings provided valuable insights toward advancing the knowledge of PARP6 in maintaining pseudo-bipolar mitosis and cancer development. To further the understanding of PARP6, finer mechanistic work is warranted to understand how Chk1 ADP-ribosylation impacts its phosphorylation, and subsequent activation and localization; for instance, mapping the ADP-ribosylation site(s) on Chk1 will be of interest for understanding the structural interplay between these two post-translational modifications. Furthermore, other centrosome related proteins in addition to Chk1 were identified as PARP6 substrates from the Protoarray screen, and thus it would be worthwhile to investigate their potential involvement in a PARP6-mediated mitotic phenotype. Finally, future work around biomarkers predicting sensitivity to AZ0108 or other PARP6 inhibitors will be crucial for identifying a patient population that will benefit from PARP6 inhibition. Our bioinformatic analysis revealed a negative correlation between the expression level of centrosome related proteins such as CENP-A, a key driver in centrosome clustering, and AZ0108 sensitivity, suggesting a future research direction to substantiate a patient enrichment strategy for a PARP6 inhibitor.
